# Relationship Between Pregnancy Complications and Serum Pregnancy Associated-Plasma-Protein-A and Free-β-Human Chorionic Gonadotropin in the First Trimester Among Iranian Women 

**Published:** 2017-12

**Authors:** Hatav Ghasemi-Tehrani, Arezoo Sadeghian, Reza Entezari

**Affiliations:** 1 Department of Obstetrics and Gynecology, School of Medicine, Isfahan University of Medical Sciences, Isfahan, Iran; 2 Students’ Research Center, School of Medicine, Isfahan University of Medical Sciences, Isfahan, Iran

**Keywords:** β-hCG, Complications, First Trimester, PAPP-A, Pregnancy

## Abstract

**Objective:** To evaluate the relationship between maternal serum levels of pregnancy-associated-plasma-protein-A (PAPP-A) and β-human-chorionic-gonadotropin (β-hCG) and pregnancy complications.

**Materials and methods:** This is a case-control study conducted during 2015- 2016. Women at their first pregnancy were enrolled and serum PAPP-A and free-β-hCG were measured in 9-14 weeks of gestation. They were followed till the end of pregnancylooking for complications including preterm labor, pregnancy induced hypertension (PIH), abortion, and intra-uterine growth retardation (IUGR).

**Results:** A total of 1070 pregnant women (mean age: 28.64 ± 4.95 years) were enrolled. Low serum levels of PAPP-A were more frequent in patients developing IUGR (17.4% versus 1.2%, p < 0.001), preterm labor (13% versus 2%, p = 0.013), and PIH (17.4% versus 4%, p = 0.015) compared those without complications. The serum levels of free-β-hCG were not different in patients with and without complications (p > 0.05).

**Conclusion:** There is an association between low serum levels of PAPP-A and developing IUGR, preterm labor, and PIH.

## Introduction

Today, one of the most effective methods for screening chromosomal abnormalities during pregnancy is a combination of fetal nuchal translucency and evaluation of maternal serum free human Chorionic Gonadotropin (β-hCG) and Pregnancy Associated Plasma Protein A (PAPP-A) through 9-14 weeks of gestation (1, 2). Furthermore, PAPP-A and β-hCG are suggested to be able to predict some pregnancy complications and some previous researchers have tried to evaluate their predicting value (3, 4).

Several studies have suggested that lower serum levels of PAPP-A in the first trimester is associated with more risk of developing pregnancy complications such as low birth weight (5), preterm labor (6), still birth (7), abortion (8), and pregnancy induced hypertension (9). Similar association has been evaluated between pregnancy complications and decreased serum level of β-hCG, however the only suggested association was found for higher risk of developing abortion in these patients (10).

Despite all these findings, there is still conflicting data on this issue in the literature (1, 4). Previous studies have limitations such as small sample sizes, presence of multiple cofounders, and incomplete exclusion criterion which makes the results unreliable. Also, most studies were conducted in developed countries, specifically European and American countries. To eliminate these limitations, we decided to design a study with a large sample size, determine rigid inclusion and exclusion criterion to eliminate any confounding factor, and follow subjects closely looking for pregnancy complications. To our knowledge, this is the first study performed in Iran and surrounding countries in Middle East on this topic. In this study we evaluate the possible association between maternal serum levels of PAPP-A and free β-hCG and some pregnancy complications.

## Materials and methods

This is a case control study which was performed during 2015 to 2016 in two obstetrics and gynecology centers (Alzahra hospital and Shahid Beheshti hospital) affiliated to Isfahan University of Medical Sciences and located in the city of Isfahan, Iran. Pregnant women with the following criteria were invited to enter the study: women who were experiencing their first pregnancy; body mass index (BMI) in the normal range (18.5-24.9). Among them, those with the following characteristics were excluded: taking immunosuppressive, corticosteroid, or thyroid hormones medication; current or previous smoking; diabetes; hypertension; and having anomalies. Also, patients were asked to deliver at our institution to be included in the study. The study was approved by the regional bioethics committee of Isfahan University of Medical Sciences (approval code: 394024) and all patients were asked to fill informed consent before entering the study.

To calculate the sample size, we used the following equation: n=Z1-α22P1-Pd2. α or Type 1 error was assumed 0.05, P was adopted from the decreased amount of PAPP-A or β-hCG and was assumed 0.5 to reach the largest possible amount of sample size, and d was the estimation error which was assumed 0.03. The sample size was calculated to be 1067 after placement of numbers.

Blood samples were taken between 9-14 weeks of gestation to measure maternal serum PAPP-A and free β-hCG using Cobas E 411 analyzer (made by Roche company, Germany, 2011). Patients were observed then till the end of pregnancy and 24 hours after that for possible complications including preterm labor (spontaneous delivery before 37^th^ week of gestation), pregnancy induced hypertension (systolic blood pressure ≥ 140 or diastolic blood pressure ≥ 90, recorded more than 1 time), abortion (delivery of dead fetus before 20^th^ week of gestation), and intra-uterine growth retardation (IUGR) (fetus estimated weight under 10 percentile based on ultrasound examination. It should be noted that these outcomes were investigated separately in each patient.

To find the association between maternal serum levels of PAPP-A and free β-hCG, and pregnancy complications we compared means of PAPP-A and β-hCG between groups of cases who developed complications and those experiencing no complications. Also, serum levels of PAPP-A and β-hCG were classified into two groups of normal range (PAPP-A ≥ 0.4 and β-hCG ≥ 0.5) and abnormal range (PAPP-A ˂ 0.4 and β-hCG ˂ 0.5) and the frequency of each group was compared among patients with and without pregnancy complications. It should be noted that these cutoffs were adopted from laboratory classic reference ranges based on multiple of medians of PAPP-A and free β-hCG serum levels in the normal population and after required corrections, and no statistical method was used to determine these cutoffs.

Data were evaluated using Kolmogorov-Smirnov test and the distribution was normal. To analyze and report data, we used descriptive statistics for reporting frequencies and means, and analytical statistics including t-test, chi-square test, ROC curve, and Pearson correlation for statistical analyses using SPSS 20 and the P-value less than 0.05 was considered as significant.

## Results

A total 1104 patients entered the study; however 34 of them left the study or did not cooperate for follow-up. Therefore, a total of 1070 patients consisted the study population with the mean age of 28.64 ± 4.95. Descriptive data on PAPP-A and β-hCG serum levels and complications of pregnancy are summarized in Table 1.

Table 2 demonstrates results from comparison of PAPP-A and free β-hCG serum levels between patients with and without complications of pregnancy. We found lower mean serum level of PAPP-A in patients with pregnancy complications, however the difference was only statistically significant in the category of preterm labor (p = 0.025). 

**Table 1:** Demographic data

**Category**	**Subcategory**	**N**	**Range**	**Mean ± SD**
Age		1070	17-44	28.64 ± 4.94
PAPP-A		1070	0.22-6	1.26 ± 0.58
	< 0.4	23		
	≥ 0.4	1047		
Β-HCG		1070	0.22-9.43	1.51 ± 1.04
	< 0.5	72		
	≥ 0.5	998		
IUGR	No	1053		
	Yes	17		
Preterm Labor	No	1046		
	Yes	24		
HTN	No	1024		
	Yes	46		
Abortion	No	1067		
	Yes	3		

SD: Standard deviation; IUGR: Intra-uterine growth retardation; HTN: pregnancy induced hypertension; PAPP-A: pregnancy associated plasma protein A; b-hcg: b human chorionic gonatitropin

Also, the mean serum level of free β-hCG was seen to be lower in patients with pregnancy complications (except those with IUGR), although none of the differences were statistically significant (p > 0.05).

As we mentioned before, we also used cutoff points for PAPP-A and β-hCG to categorize them into two groups of low and normal range, and compared the frequency of each group between patients with and without pregnancy complications. These results are shown in Table 3. PAPP-A < 0.4 was more frequent in patients who developed IUGR, preterm labor, and pregnancy induced hypertension (17.4% versus 1.2%, P<0.001; 13% versus 2%, p = 0.013; and 17.4% versus 4%, p = 0.015 respectively). On the other hand, the frequency of β-hCG < 0.5 and β-hCG ≥ 0.5 among patients with and without pregnancy complications showed a non-significant difference between any of the groups (p > 0.05). It is noteworthy that results from abortion can’t be judged because of very small number of cases in this group. Pearson correlation showed a direct relationship between β-hCG and PAPP-A (r = 0.195, p-value < 0.001).

## Discussion

In this study we evaluated the association between decreased maternal serum levels of PAPP-A and free β-hCG, and some pregnancy complications (IUGR, preterm labor, pregnancy induced hypertension, and abortion).

PAPP-A is chorionic product which is released in maternal blood during pregnancy. This protein increases in maternal serum during pregnancy and decreases rapidly after delivery (11).

**Table 2:** Comparison of PAPP-A and Β-HCG means between complications of pregnancy

**Category**		**PAPP-A (mean (SD))**	**P-value**	**Β-HCG (mean (SD))**	**P-value**
IUGR	Yes	1.07 (0.57)	0.184	1.52 (1.22)	0.981
	No	1.26 (0.58)		1.51 (1.03)	
Preterm labor	Yes	1.02 (0.48)	0.025	1.23 (0.69)	0.058
	No	1.26 (0.58)		1.52 (1.04)	
HTN	Yes	1.21 (0.69)	0.647	1.40 (1)	0.443
	No	1.26 (0.58)		1.52 (1.04)	
Abortion	Yes	0.75 (0.46)	0.193	0.86 (0.90)	0.334
	No	1.26 (0.58)		1.51 (1.04)	

PAPP-A: Pregnancy associated plasma protein A; B-HCG: B human chorionic gonadotrophin; SD: Standard deviation; IUGR: intra-uterine growth retardation; HTN: pregnancy induced hypertension

**Table 3:** Comparison of frequency of pregnancy complications between cutoff points of PAPP-A and Β-HCG

**Category**		**PAPP-A < 0.4**	**PAPP-A ≥ 0.4**	**P-value**	**OR**	**CI**	**Β-HCG < 0.5**	**Β-HCG ≥ 0.5**	**P-value**	**OR**	**CI**
IUGR	Yes	4 (17.4%)	13 (1.2%)	< 0.001	16.74	4.99, 56.10	1 (1.4%)	16 (1.6%)	0.681	0.864	0.11, 6.61
	No	19 (82.6%)	1034 (98.8%)				71 (98.6%)	982 (98.4%)			
Preterm labor	Yes	3 (13%)	21 (2%)	0.013	7.32	2.02, 26.57	3 (4.2%)	21 (2.1%)	0.215	2.02	0.58, 6.94
	No	20 (87%)	1026 (98%)				69 (95.8%)	977 (97.9%)			
HTN	Yes	4 (17.4%)	42 (4%)	0.015	5.03	1.64, 15.46	6 (8.3%)	40 (4%)	0.082	2.17	0.89, 5.32
	No	19 (82.6%)	1005 (96%)				66 (91.7%)	958 (96%)			
Abortion	Yes	1 (4.3%)	2 (0.2%)	0.063	23.75	2.07, 271.75	2 (2.8%)	1 (0.1%)	0.013	28.46	2.55, 318
	No	22 (95.7%)	1045 (99.8%)				70 (97.2%)	997 (99.9%)			

PAPP-A: Pregnancy associated plasma protein A; B-HCG: B human chorionic gonadotrophin; SD: Standard deviation; IUGR: intra-uterine growth retardation; HTN: pregnancy induced hypertension; OR: Odds ratio; CI: Confidence interval

This factor is used routinely for screening of Down syndrome during the first trimester and its decreased serum levels can show evidences of chromosomal abnormalities (11). The predicting value of PAPP-A for pregnancy complications has been addressed in several studies before (12). Previous studies have addressed low birth weight to be more associated with low maternal serum PAPP-A (13, 14). In spite of all that, some studies have reported no association between PAPP-A serum levels and risk of low birth weight (15). Although we found lower but not significant mean PAPP-A in serum of patients developing IUGR, the frequency of low range PAPP-A was significantly higher in these patients.

Data on the relationship between PAPP-A serum levels and preterm labor is conflicting in the literature (1, 16), however few studies have drawn an association between low maternal serum PAPP-A and chance of preterm delivery (10, 17). In this study, we found that mean serum levels of PAP-A is lower in patients who experienced preterm delivery later (1.02 versus 1.26). Also, the frequency of serum levels of PAPP-A in low range was significantly higher among patients who developed preterm labor.

Decreased serum levels of PAPP-A in patients developing pregnancy induced hypertension have been reported in several studies previously (13, 17, 18). Low serum levels of PAPP-A may show inadequate or impaired placentation which leads to developing pregnancy induced hypertension (19). This can explain why PAPP-A may be associated with pregnancy induced hypertension. Although we found no difference between mean PAPP-A in patients with and without pregnancy induced hypertension, the frequency of low range PAPP-A was higher in patients developing pregnancy induced hypertension.

Miscarriage has been suggested to be associated with decreased maternal serum levels of PAPP-A in the first trimester in few studies (1, 9, 20). PAPP-A serum level is used for screening chromosomal abnormalities, like Down syndrome, and is decreased in them (11). On the other hand, many pregnancies with chromosomal abnormalities may lead to miscarriage (11). We found that the average of serum levels of PAPP-A is non-significantly lower in patients who developed miscarriage. Also, the frequency of PAPP-A in the abnormal range was smaller in patients developing miscarriage, but still statistically non-significant. Therefore, our results don’t confirm previous findings on the association of decreased PAPP-A serum levels and miscarriage, however, considering the very small number of miscarriage cases in our study, our results on this category may not be reliable. 

β-hCG is a hormone excreted from chorionic villus syncytiotrophoblast during pregnancy, and induces progesterone release by stimulating receptors of luteinizing hormone and hCG in corpus luteum (21). Maternal serum free β-hCG is measured for various clinical reasons such as diagnosis of pregnancy, some pregnancy complications, screening chromosomal abnormalities, and screening gynecological cancers (22). 

The predicting role of serum free β-hCG in the first trimester for pregnancy complications is studied before, but few of them have suggested such associations between low serum free β-hCG and pregnancy complications (10). In a study researchers measured serum levels of free β-hCG in 10-14 weeks of gestation in 5584 patients and followed them until the end of pregnancy. They found approximately 15% of patients who experienced abortion, hypertension, growth disorder, and gestational diabetes had serum free β-hCG levels below 10 percentile suggesting that β-hCG is associated with pregnancy complications (1).

Another study evaluated 34271 patients in 10-14 weeks of pregnancy for PAPP-A and β-hCG serum levels and nuchal translucency. They found that patients with free β-hCG serum levels under 1 percentile developed abortion more in < 24 weeks (17). Furthermore, a recent study on 427 patients showed no association between serum levels of PAPP-A and β-hCG in the first trimester and pregnancy complications suggesting that these factors are not associated with pregnancy complications (23).

We found no statistically significant difference between the average of maternal serum free β-hCG between patients with and without pregnancy complications, however these results were close to significant levels for preterm labor. Moreover, comparing the frequency of low range and normal range β-hCG between pregnancy complications resulted in no significant finding, except for abortion. In spite of all that, because of the small number of patients with miscarriage this finding can’t be judged. Therefore, our results suggest no association between β-hCG and pregnancy complications, except for abortion, which needs to be investigated on more patients.

In this study we had some limitations that should be mentioned. First, considering the small number of abortions, we would have more precise results if we enrolled larger number of cases in the study. Second, we evaluated four pregnancy complications, limiting our findings to these complications only. Third, this study was designed as a case-control study, which has its own limitations and biases.

To conclude, PAPP-A and β-hCG are measured in many pregnant women in the first trimester for evaluation of possible chromosomal disorders. Therefore, investigating their association with pregnancy complications is beneficial. In this study we found that low serum levels of PAPP-A are associated with developing IUGR, preterm labor, and pregnancy induced hypertension, however, low serum free β-hCG is only associated with abortion.

## Conclusion

In this study we found that there is an association between low serum levels of PAPP-A and developing IUGR, preterm labor, and pregnancy induced hypertension, however, low serum free β-hCG may be associated with abortion only.
